# Residual Kidney Function and Response of Left Ventricular Mass to Intensive Hemodialysis: The Frequent Hemodialysis Network Trials

**DOI:** 10.34067/KID.0000000987

**Published:** 2025-09-23

**Authors:** Amin Tajerian, Tariq Shafi, Jason Zhang, Sobia Khan, Sandeep K. Mallipattu, Andreas P. Kalogeropoulos

**Affiliations:** 1 Baylor Scott and White Research Institute, Dallas, Texas; 2 Division of Nephrology, Baylor Scott and White Health, Temple, Texas; 3 Renaissance School of Medicine, Stony Brook University, Stony Brook, New York; 4 Division of Nephrology and Hypertension, Department of Medicine, Renaissance School of Medicine, Stony Brook University, Stony Brook, New York; 5 Division of Nephrology, Department of Medicine, Northport Veterans Affairs Medical Center, Northport, New York; 6 Division of Cardiology, Department of Medicine, Renaissance School of Medicine, Stony Brook University, Stony Brook, New York

**Keywords:** cardiovascular disease, chronic hemodialysis, dialysis, ESKD, gender difference, hemodialysis, hemodialysis adequacy, left ventricular hypertrophy, outcomes

## Abstract

**Key Points:**

- Residual kidney function does not modify the response of left ventricular mass (LVM) to frequent hemodialysis in patients with ESKD.
- Both anuric and nonanuric patients showed similar improvements in LVM with intensive hemodialysis.
- Changes in residual kidney function were not associated with response of LVM to frequent hemodialysis.

**Background:**

Frequent (6 times/wk) hemodialysis has been shown to reduce left ventricular mass (LVM) more than conventional (3 times/wk) hemodialysis in patients with ESKD. Previous work suggested that the LVM benefits of frequent hemodialysis might be attenuated in the presence of residual kidney function (RKF). We hypothesized that patients without RKF would have greater LVM reduction with frequent hemodialysis compared with patients with RKF.

**Methods:**

We analyzed data from the Frequent Hemodialysis Network (FHN) Daily (in-center) and Nocturnal (home-based) trials, which randomized patients with ESKD to either frequent or conventional hemodialysis over 12 months. RKF was evaluated through timed 24-hour urine collections, with anuria defined as urine output <100 ml/day. LVM was assessed through magnetic resonance imaging at baseline and 12 months. Combined trial data were analyzed using log-transformed LVM to evaluate the effect of RKF on LVM change.

**Results:**

Among 332 patients from both FHN trials (170 frequent, 162 conventional hemodialysis), 189 (57%) were anuric at baseline. Baseline LVM did not differ between anuric and nonanuric patients (log LVM: 4.90±0.32 versus 4.85±0.40; *P* = 0.18). At 12 months, both anuric and nonanuric patients experienced similar LVM reductions with frequent hemodialysis (mean change in log LVM: −0.11±0.23 versus −0.08±0.23; *P* = 0.44). No association was found between baseline urine volume and LVM response (rho=−0.002, *P* = 0.98 for frequent; rho=0.003, *P* = 0.97 for conventional hemodialysis). Changes in urine volume over 12 months did not correlate with LVM changes. In an exploratory analysis, LVM reduction correlated with urine volume decline in men (rho=0.23; *P* < 0.01) but not in women (rho= −0.19; *P* = 0.10; *P*_interaction_ = 0.003).

**Conclusions:**

Both anuric and nonanuric patients experienced similar LVM reductions with frequent hemodialysis, and no association between urine output and LVM response was observed in the FHN trials. These data suggest that the response of LVM to frequent hemodialysis is independent of baseline RKF.

## Visual Abstract

**Figure:**
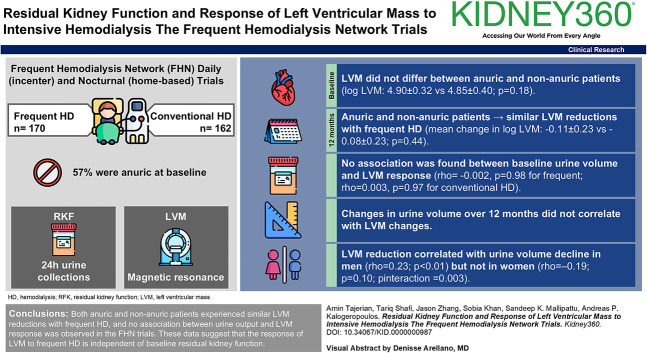

## Introduction

Cardiovascular disease accounts for over 50% of mortality among patients with ESKD.^1^ Left ventricular hypertrophy (LVH), an indicator of the adaptive responses to factors such as hypertension, hypervolemia, and anemia,^2^ is common among patients with CKD before ESKD and continues to worsen with CKD progression and ESKD.^3,4^ Importantly, LVH is a key contributor to cardiovascular disease and mortality in CKD, particularly among patients receiving hemodialysis for ESKD.^3,5^

Previous studies have shown that changes in left ventricular mass (LVM) independently predict cardiac outcomes in ESKD^2^ and that intensive hemodialysis schedules, such as short daily or nocturnal treatments, can significantly reduce LVM and improve cardiovascular and heart failure–related outcomes.^2,6,7^ The Frequent Hemodialysis Network (FHN) trials^7,8^ randomized patients with ESKD on dialysis to either 12 months of conventional three times per week daily hemodialysis or frequent six times per week hemodialysis, either in-center during the day (Daily trial) or at night (Nocturnal trial) at home. In the Daily trial, a significant reduction in LVM after 12 months of frequent hemodialysis was observed.^7^ Subsequent analysis evaluating data from the Daily and Nocturnal trials separately suggested that this effect was more pronounced among patients with negligible urine output at baseline and that residual kidney function (RKF) may influence the effect of intense hemodialysis on LVM, although individual interactions did not reach statistical significance in individual trial analyses.^6^

RKF is associated with improved dialysis adequacy and better survival among patients with ESKD undergoing hemodialysis.^9,10^ Patients with preserved RKF demonstrate better volume control and uremic toxin clearance than anuric patients, contributing to improved fluid balance and BP control, which in turn can mitigate the progression of LVH in patients with ESKD,^10,11^ potentially explaining the favorable effect of RKF on survival in these patients. Conversely, loss of RKF contributes to fluid overload, hypertension, and toxin accumulation, which are drivers of LVH in ESKD.^6,12^ Therefore, the presence of RKF may potentially diminish the effects of intensive dialysis interventions on LVH progression. Further analysis of combined data from the FHN trials could provide additional insights into the influence of RKF on LVM response to frequent hemodialysis.

In this work, we combined data from the FHN trials to comprehensively evaluate the effect of RKF on the effect of frequent hemodialysis on LVM. We hypothesized that patients without RKF would experience more reductions in LVM with frequent hemodialysis compared with those with preserved RKF because of their greater dependence on dialysis for volume and toxin clearance and thus a larger potential for cardiovascular improvement through intensified treatment.

## Methods

### Study Population

We analyzed data collected in the FHN Daily and Nocturnal trials, both multicenter randomized clinical trials. The Daily trial compared the efficacy of six times per week in-center (frequent) versus three times per week in-center (conventional) hemodialysis for 12 months. The Nocturnal trial compared six times per week home nocturnal hemodialysis to three times per week conventional home hemodialysis for 12 months. The Daily trial assigned 125 patients to frequent and 120 to conventional hemodialysis, whereas the Nocturnal trial assigned 45 and 42 patients to frequent and conventional hemodialysis, respectively. Patients were enrolled between March 2006 and May 2009. Patients were excluded from the Daily trial if urea clearance was >3 ml/min per 35 L and from the Nocturnal trial if the average of the urea and creatinine clearance was >10 ml/min per 1.73 m^2^.^6,8^ The complete inclusion and exclusion criteria of the FHN trials have been previously described.^6,8,12–14^ All subjects from both FHN Daily and Nocturnal trials were included in the present analysis. This analysis was approved by the Institutional Review Board of Stony Brook University.

### RKF

RKF was measured through timed urine collections. Time-averaged concentrations of urea and creatinine were measured to calculate solute clearance from urine collected over at least 18 hours for patients in the Daily trial and 24 hours for patients in the Nocturnal trial. Urine collections were obtained during the interdialytic interval preceding a dialysis session. In both the Daily and Nocturnal groups, urine and blood samples were collected at baseline and again at 4 months and 12 months of follow-up for patients with non-negligible urine production defined as follows. In the FHN daily trial, the presence of RKF was defined as producing at least 80 ml of urine volume with renal urea clearance <3 ml/min per 35 L. Patients in the Daily trial who produced <80 ml of urine were considered anuric. In the FHN Nocturnal trials, the presence of RKF was defined as producing at least 100 ml of urine, with renal urea clearance <10 ml/min per 1.73 m^2^ measured by the average of the urea and creatinine clearances. Patients in the Nocturnal trial who produced <100 ml of urine were considered anuric.

For the present analyses, RKF was assessed using 24-hour urine volume, with anuria defined as urine output <100 ml/day. This standardized cutoff was applied across both the FHN Daily and Nocturnal trials to reduce variation within the anuric group and to ensure analytical consistency, as only two patients in the daily trial had urine volumes between 80 and 100 ml. Urine volume was selected as the primary measure because it is a practical, widely available, and clinically relevant indicator of RKF commonly used in routine dialysis care.

### LVM

The primary end point of interest in this analysis was LVM, measured using cardiac magnetic resonance imaging at baseline and 12 months. Standard protocols and technical details for the measurement and calculation of ventricular mass have been previously described.^6,7^

### Data Analysis

We combined data from the FHN Daily and Nocturnal trials to evaluate the effect of RKF on LVM change over a 12-month period. Because LVM followed a log-normal distribution, we log-transformed LVM (log-LVM) for inferential analyses. We compared baseline LVM between groups of interest and changes in LVM (from baseline to 12 months) within groups using unpaired and paired Student *t* tests, respectively. We also compared the changes in LVM between groups of interest using unpaired *t* test on the change. Change in urine volume was calculated as the difference between 12-month and baseline 24-hour urine volumes for patients with available data at both time points. For patients who became anuric during the study period, the 12-month value was recorded as 0 ml. Square-root transformation was applied to the urine volume to satisfy normality assumptions for correlation analyses. We conducted *post hoc* exploratory subgroup analyses to assess whether age, sex, or race modified the relationship between changes in urine output and LVM response to intensive hemodialysis. For all analyses, we considered a *P* value ≤ 0.05 as statistically significant. All statistical analyses were performed using R version 4.4.1 in Posit Cloud (Posit Software, Boston, MA).

## Results

### Study Population

The FHN Daily trial randomized 245 patients to frequent versus conventional in-center hemodialysis, of which 33% were anuric (81/245). The FHN Nocturnal trial randomized 87 patients to frequent nocturnal versus conventional home hemodialysis, of which 71% were anuric (62/87). The baseline 24-hour urine volume among patients with RKF, presented as median (interquartile range [IQR]), was significantly different between the Daily and Nocturnal trials. In the Daily trial, the median volume output was 400 ml (IQR, 221.5–600.0 ml), while in the Nocturnal trial, it was 711.1 ml (IQR, 427.7–997.1 ml). The median baseline LVM was 130.8 g (range 34.7–360.8 g) and 132.8 g (range 47–257.3 g) in the Daily and Nocturnal trials, respectively. Baseline demographics, clinical characteristics, medication use, biochemical profile, and outcomes of interest are summarized in Table 1.

**Table 1:** Baseline characteristics of patients according to baseline urine output status

Characteristics	Baseline Urine Output		*P* Value^b^
	RKF+≥100 ml/24 h (*N*=143)^a^	RKF−<100 ml/24 h (*N*=189)^a^	
**Study characteristics**			
FHN study			<0.001
Daily	81 (56.6%)	164 (86.8%)	
Nocturnal	62 (43.4%)	25 (13.2%)	
Randomization group for treatment			0.076
Frequent hemodialysis (six times/wk)	65 (45.5%)	105 (55.6%)	
Conventional hemodialysis (three times/wk)	78 (54.5%)	84 (44.4%)	
Yr on dialysis	1.2 (0.5–3.3)	5.3 (2.6–9.0)	<0.001
Urine volume in 24 hours (ml)	500.0 (275.0–793.0)	0.0 (0.0–0.0)	N/A
**Demographics**			
Age	53.3 (43.2–63.4)	49.7 (41.6–58.1)	0.051
Sex: male	97 (67.8%)	111 (58.7%)	0.11
Race			0.3
Asian	14 (9.8%)	14 (7.4%)	
Black, African American, African	46 (32.2%)	79 (41.8%)	
Other, unknown, or not reported	18 (12.6%)	24 (12.7%)	
White/Caucasian	65 (45.5%)	72 (38.1%)	
Hispanic	18 (12.6%)	51 (27.0%)	0.002
BMI	27.5 (23.6–33.5)	25.2 (21.8–32.7)	0.023
**Comorbidities**			
Atrial fibrillation	5 (3.5%)	15 (7.9%)	0.11
Myocardial infarction	16 (11.2%)	20 (10.6%)	0.9
Congestive heart failure	21 (14.7%)	40 (21.2%)	0.2
Connective tissue disease	3 (2.1%)	12 (6.3%)	0.11
Peripheral vascular disease	17 (11.9%)	23 (12.2%)	>0.9
Cerebrovascular accident	9 (6.3%)	11 (5.8%)	>0.9
Chronic pulmonary disease	7 (4.9%)	8 (4.2%)	0.8
Diabetes	66 (46.2%)	71 (37.6%)	0.14
Liver disease	7 (4.9%)	12 (6.3%)	0.6
Alcohol use			0.6
No history	126 (88.1%)	171 (90.5%)	
Yes, currently	0 (0.0%)	1 (0.5%)	
Yes, used to drink in excess	17 (11.9%)	17 (9.0%)	
Smoking history			0.5
Currently smokes	18 (12.6%)	33 (17.5%)	
Never smoked	88 (61.5%)	110 (58.2%)	
Used to smoke	37 (25.9%)	46 (24.3%)	
**Kidney failure characteristics**			
Kidney failure etiology			0.002
Diabetic nephropathy	58 (40.6%)	56 (29.6%)	
Glomerulonephritis	38 (26.6%)	40 (21.2%)	
Hypertensive nephrosclerosis	18 (12.6%)	40 (21.2%)	
Other or unknown	20 (14.0%)	49 (25.9%)	
Polycystic kidney disease	9 (6.3%)	4 (2.1%)	
Peritoneal dialysis	12 (8.4%)	32 (16.9%)	0.033
Medications			
IV Iron total dose (mg)	1950 (1,250–2875)	1525 (875–2200)	<0.001
Total erythropoietin units (4 wk)	74,100 (0–186,800)	96,000 (24,000–226,800)	0.062
Total vitamin D dose (1 wk)	12.0 (0.0–42.0)	24.0 (6.0–57.0)	0.002
ACE inhibitors	40 (28.0%)	61 (32.3%)	0.5
Antihypertensives	130 (90.9%)	158 (83.6%)	0.071
ARBs	28 (19.6%)	39 (20.6%)	0.9
*β* blockers	90 (62.9%)	109 (57.7%)	0.4
Diuretics	35 (24.5%)	15 (7.9%)	<0.001
Statins	76 (53.1%)	59 (31.2%)	<0.001
Vasodilators	10 (7.0%)	27 (14.3%)	0.052
**Laboratory results**			
Hemoglobin	11.9 (11.4–12.5)	11.9 (11.3–12.7)	0.7
Unknown	5	8	
Transferrin	26.0 (20.0–32.0)	28.0 (22.0–38.0)	0.055
Ferritin	441.0 (246.0–727.0)	545.0 (336.5–777.0)	0.053
TIBC	226.0 (196.0–273.0)	211.0 (183.0–227.0)	0.2
Unknown	132	170	
Corrected calcium	9.1 (8.8–9.5)	9.1 (8.7–9.7)	0.7
Monthly albumin	4.0 (3.6–4.3)	4.0 (3.7–4.3)	0.6
Unknown	49	85	

ACE, angiotensin converting enzyme; ARB, angiotensin receptor blockers; BMI, body mass index; FHN, Frequent Hemodialysis Network; IV, intravenous; RKF, residual kidney function; TIBC, total iron binding capacity.

a Median (25th percentile, 75th percentile) or frequency (%)

b Fisher exact test; Wilcoxon rank-sum test

### Baseline RKF and LVM

**Figure 1:**
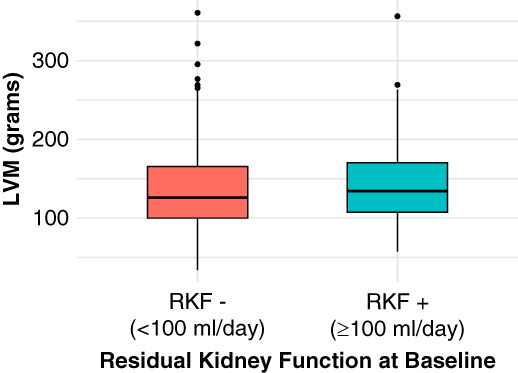
**LVM at baseline, stratified by RKF at baseline.** LVM, left ventricular mass; RKF, residual kidney function.

**Figure 2:**
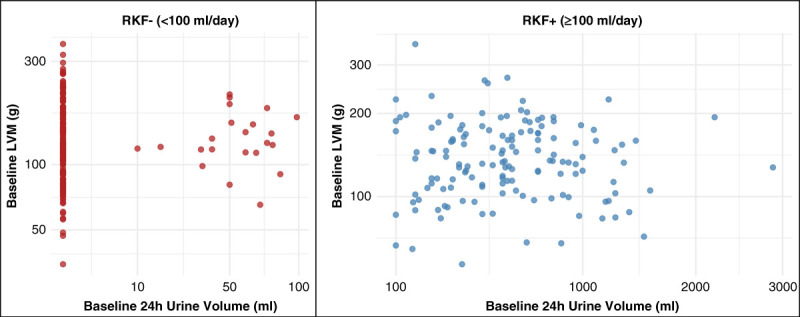
**Correlation between LVM at baseline and urine volume at baseline.** Rho=0.067 (*P* = 0.22) in the entire cohort; rho=−0.004 (*P* = 0.96) in the non-anuric patients. Note that the *x* axis is on the square-root scale and the *y* axis is on the log scale.

Across the Daily and Nocturnal trials, anuric patients and patients with RKF (nonanuric) did not have significantly different baseline LVM (Figure 1). Anuric patients had slightly greater LVM than nonanuric patients, but this difference was not statistically significant (log-LVM: 4.90±0.32 versus 4.85±0.40; *P* = 0.18). In continuous urine volume analysis, there was no association between urine volume at baseline and baseline LVM (rho=0.067; *P* = 0.22; Figure 2). Restricting the analysis to those with urine volume ≥100 ml did not change the results (rho=−0.004; *P* = 0.96).

### RKF and Response of LVM to Intense Hemodialysis

In the pooled analysis of the Daily and Nocturnal trials, anuric status at baseline had no significant effect on the response of LVM (Supplemental Table 1). Individuals with and without anuria experienced similar reductions in log-LVM with 6/wk hemodialysis (mean±SD: nonanuric=−0.08±0.23, anuric=−0.11±0.23; *P* = 0.44). For patients who received conventional 3/wk hemodialysis, the log-LVM was slightly lower in those without anuria but was not statistically significant (nonanuric=−0.03±0.18, anuric=0.004±0.19; *P* = 0.43).

In the Daily trial, patients receiving 6/wk in-center hemodialysis demonstrated a significantly greater reduction in LVM, compared with those on 3/wk in-center hemodialysis (mean change: −0.11 versus −0.03; *P* = 0.01). By contrast, in the Nocturnal study, no significant difference in log-LVM was noted between the 6/wk nocturnal home hemodialysis and 3/wk daytime home hemodialysis treatment arms (mean change: −0.07 versus +0.01; *P* = 0.11; Supplemental Figure 1). Within each trial, anuria was not associated with the response of LVM to 6/wk hemodialysis (Supplemental Table 1).

**Figure 3:**
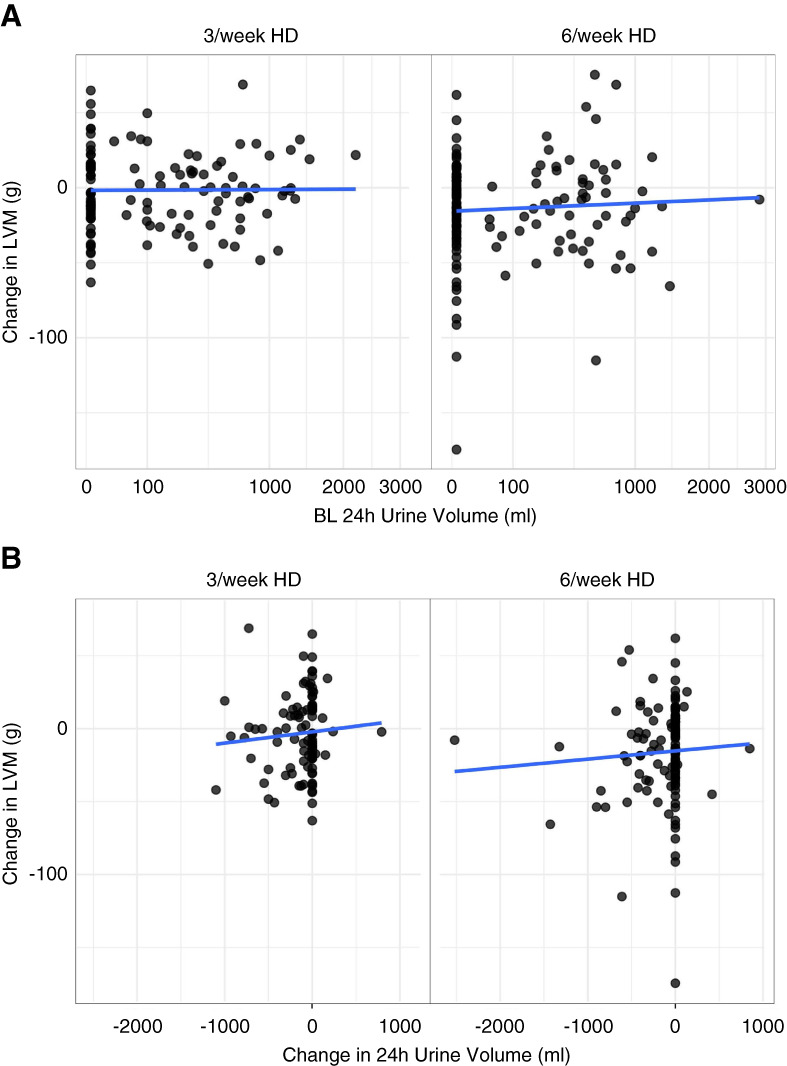
**Association of RKF with LVM response to frequent hemodialysis.** (A) Scatterplot of 24-hour urine volume at baseline and the change in LVM with 6/wk (left) or 3/wk (right) hemodialysis. Note that the *x* axis is on the square-root scale. Rho = −0.002 (*P* = 0.98) in the 3/wk hemodialysis patients; rho=0.003 (*P* = 0.97) in the 6/wk hemodialysis patients. (B) Scatterplot of change in 24-hour urine volume (square-root-transformed) and change in LVM with 6/wk (left) or 3/wk (right) hemodialysis. Rho=0.07 (*P* = 0.51) in the 3/wk hemodialysis patients; rho=0.11 (*P* = 0.23) in the 6/wk hemodialysis patients. BL, baseline; HD, hemodialysis.

When baseline urine output was evaluated as a continuous variable, there was no association between baseline 24-hour urine volume and the change in LVM with 6/wk and 3/wk hemodialysis (rho=−0.002, *P* = 0.98; rho=0.003, *P* = 0.97; Figure 3A). The association between LVM response and baseline 24-hour urine volume was not influenced by the treatment arm (*P* for interaction=0.769).

Change in 24-hour urine volume from baseline to year 1 suggested a trend toward greater reduction in LVM in those with a greater reduction in urine volume, but the results were not statistically significant (rho=0.11, *P* = 0.23; rho=0.07, *P* = 0.51; Figure 3B).

### Exploratory Analyses

**Figure 4:**
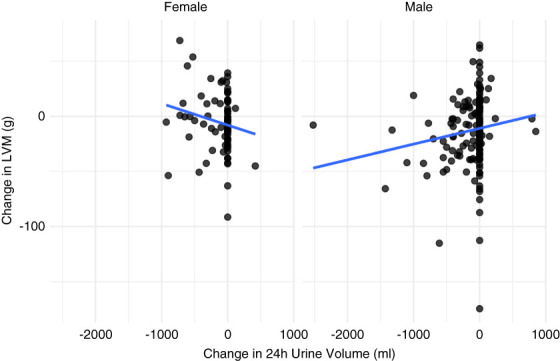
**Change in 24-hour urine output and change in LVM in male versus female participants.** Rho=0.23 (*P* < 0.01) in the male patients; rho=−0.19 (*P* = 0.10) in the female patients.

In a *post hoc* exploratory subgroup analysis, we used a multivariable linear regression model with interaction terms to assess whether demographic factors including age, sex, and race modified the association between changes in 24-hour urine output and LVM response to hemodialysis. A statistically significant interaction was found for sex (*P* for interaction = 0.003); see Figure 4. Among male patients, reductions in urine volume were associated with reductions in LVM (rho=0.23, *P* < 0.01), whereas among female patients, the association was inverse and not statistically significant (rho=−0.19, *P* = 0.10). No significant interactions were observed for age (*P* = 0.40) or race (Black: *P* = 0.97; other/unknown: *P* = 0.47; White=reference).

To assess whether the relationship between RKF and the LVM response to frequent hemodialysis could be explained by baseline differences between patient groups, we conducted an exploratory analysis adjusting for selected available covariates including age, sex, race, baseline hemoglobin, dialysis vintage, dialysis modality, and intravenous iron use. In this model, frequent hemodialysis (6/wk) remained significantly associated with reductions in LVM (*P* = 0.022). Changes in RKF were not associated with LVM change (*P* = 0.187), and there was no significant interaction between RKF change and treatment arm (*P* = 0.900).

## Discussion

This analysis represents the first comprehensive evaluation of RKF's effect on LVM response to intensive hemodialysis using the combined FHN Daily and Nocturnal trial datasets. While prior analyses of individual FHN trials^6^ provided suggestive evidence that RKF might modify LVM response, these were limited by smaller sample sizes and were not specifically powered to detect RKF-treatment interactions. Our pooled analysis, with larger sample size and standardized RKF definitions across both trials, provides definitive evidence that RKF does not significantly modify the cardiovascular benefits of intensive hemodialysis. This finding resolves previous uncertainty and provides clear guidance for clinical decision making regarding intensive dialysis prescriptions.

We observed that, while in accordance with previous findings frequent hemodialysis was associated with significant reductions in LVM, this effect did not differ significantly between patients with RKF and those who were anuric at baseline, nor was the LVM response correlated with baseline urine volume as a continuous variable. Similarly, changes in urine volume over the course of treatment did not correlate with LVM changes, indicating that neither baseline anuric status nor urine volume influenced the effect of intense hemodialysis on LVM. Several findings in this study merit further discussion.

Although other factors such as BP control, anemia management, and interdialytic fluid gains may mediate the relationship between intensive dialysis and LVM changes, our analysis deliberately focused on RKF as the primary variable of interest. Including additional mediating variables in the statistical models would likely attenuate the observed associations and could mask the specific effect of RKF that we sought to investigate. Our approach allows for a focused assessment of whether RKF independently modifies the LVM response to intensive dialysis. However, we acknowledge that other baseline characteristics could influence RKF and LVM change relationship. In an exploratory analysis adjusting for selected covariates, the results were unchanged: frequent hemodialysis remained significantly associated with LVM reduction, RKF was not associated with LVM change, and did not mediate the treatment effect. Future studies with larger sample sizes might explore more complex mediation analyses incorporating multiple pathways, but such analyses were beyond the scope of our current investigation.

The potential effect of RKF on LVH was first assessed in patients undergoing peritoneal dialysis, where a decrease in RKF was associated to increased LVH severity, poorer cardiac function, and worse outcomes. Conversely, preserved RKF was associated with decreased LVH, higher hemoglobin and serum albumin levels, and improved dialysis adequacy.^15,16^ In addition, in patients with ESKD undergoing long-term continuous ambulatory peritoneal dialysis, a decline in RKF was associated with increased LVM, suggesting that loss of RKF contributes to the worsening of LVH.^15,16^ However, the contribution of RKF to LVM changes in hemodialysis patients has not been thoroughly examined. In a *post hoc* analyses of the FHN data, eight of nine patients who experienced a reduction in LVM of 60 g or more had negligible residual urine volume^6^ and among patients with RKF at baseline, the reduction in LVM did not achieve statistical significance in either the Daily or Nocturnal trials.^6^ These findings have led to speculations that RKF may be a significant modifier of LVM response to intense hemodialysis. However, contrary to this hypothesis, our detailed analysis using both dichotomous definitions and continuous variable analysis indicate that baseline RKF status and changes in urine output are not associated with changes in LVM in response to hemodialysis.

The absence of a significant RKF effect on LVM response to intensive hemodialysis may reflect fundamental differences in the pathophysiology of cardiac remodeling in peritoneal dialysis versus hemodialysis patients. Although RKF preservation in peritoneal dialysis patients contributes to better volume control and uremic toxin clearance over continuous 24-hour cycles, the intermittent nature of hemodialysis creates distinct hemodynamic stresses that may override any protective effects of RKF. Major fluid shifts, rapid electrolyte changes, and cyclical volume loading and unloading inherent to hemodialysis may all represent stimuli for cardiac remodeling that are disproportionately potent compared with the more subtle benefits of preserved RKF. Furthermore, the mechanisms underlying LVM reduction with intensive hemodialysis likely operate through pathways that are independent of RKF. Intensive hemodialysis regimens improve phosphorus clearance, reduce interdialytic fluid accumulation, and provide better BP control through enhanced volume management benefits that accrue regardless of baseline urine output.^17^ The removal of middle molecules and protein-bound uremic toxins with more frequent dialysis sessions may also contribute to reduced inflammatory burden and improved cardiac metabolism,^18^ effects that would be expected to benefit all patients irrespective of RKF status. Another consideration is that patients with RKF may have had smaller baseline LVM, potentially creating a “floor effect” where further reductions become more difficult to achieve or detect. Prior work from the FHN trials suggested that the effect of frequent hemodialysis on LVM was more pronounced among patients with higher baseline LVM.^6^ However, our data do not support this hypothesis, as baseline LVM did not differ significantly between anuric and nonanuric patients. This suggests that by the time patients reach dialysis initiation, the cardiac consequences of uremia may be sufficiently advanced that RKF no longer provides meaningful cardio-protection, at least for structural cardiac changes. Finally, few patients with RKF were enrolled in the FHN trials, and it is reasonable to consider that the LVM response among patients with RKF may not have been significant due to a combination of attenuated effect and inadequate power.

An exploratory finding from our analysis was a significant sex-based difference in how changes in urine volume correlated with LVM response. Among male patients, reductions in 24-hour urine volume were associated with reductions in LVM, while among female patients, the correlation was inverse and nonsignificant. This observation is consistent with previous literature demonstrating sex differences in LVH prevalence and determinants among dialysis patients. Female patients receiving dialysis have a 2.5-fold to four-fold higher risk of developing LVH compared with men^19^ and determinants of LVH differ by sex, *e.g*., pulse pressure being important in males but not women.^20^ However, given the exploratory nature of this analysis, the relatively small subgroup sizes when stratified by both sex and RKF status, and the *post hoc* design of our study, these findings should be interpreted with extreme caution. While intriguing, this sex-based interaction warrants validation in future prospective studies.

Our findings have important implications for clinical practice and dialysis prescription. The observation that RKF does not significantly modify the LVM response to intensive hemodialysis suggests that the cardiovascular benefits of frequent hemodialysis extend broadly across the dialysis population, regardless of RKF status. This is clinically reassuring, as it indicates that patients should not be excluded from consideration for intensive hemodialysis regimens based solely on their urine output. Instead, clinicians can recommend intensive hemodialysis based on other clinical factors, including difficulty achieving adequate volume control, persistent hypertension, or high cardiovascular risk, without considering RKF as a major determinant of likely cardiac benefit. This may be particularly relevant for nocturnal hemodialysis programs, where patient selection often involves complex considerations of medical suitability.

Although frequent in-center hemodialysis, as studied in the FHN Daily trial, is uncommon in current practice, intensive dialysis is primarily delivered through home-based modalities, including short Daily and Nocturnal home hemodialysis. Our results further support that RKF should not guide patient selection for these home dialysis programs. Clinicians may instead rely on clinical and psychosocial considerations, such as patient motivation, suitability of the home environment, and cardiovascular risk, without concern that preserved RKF might diminish the cardiac benefits derived from intensive dialysis.

This study has several limitations. First, this is a *post hoc* analysis using pooled data from the FHN trials, and therefore, it was not powered specifically to demonstrate differences in LVM response according to baseline urine output. Therefore, the power was limited by the existing sample size, and we cannot exclude the possibility that clinically meaningful differences in RKF effects on LVM response may have been missed due to insufficient statistical power. Second, the enrollment definitions of anuric status and RKF were different in the two trials. Although we applied the same definition for the purposes of this analysis, the differential inclusion criteria may have diluted the observed effect of RKF on LVM. Third, the relatively short follow-up period of 12 months may not be sufficient to detect longer term effects of RKF on cardiac remodeling. Fourth, RKF was assessed using timed urine collections, which can be subject to collection errors and may not fully reflect the complex contributions of RKF to overall patient physiology. While formal measurement of residual clearance (urea and creatinine) might provide a more precise assessment of kidney function, urine volume represents a clinically accessible measure that correlates well with overall RKF and is commonly used in routine dialysis care. Our analysis focused specifically on RKF and did not comprehensively examine other potential mediators of the intensive dialysis-LVM relationship, such as BP control, phosphorus management, or interdialytic weight gains. While this focused approach allows for clear interpretation of RKF effects, future studies might benefit from more comprehensive mediation analyses. Finally, the FHN trial populations may not be representative of the broader dialysis population, as FHN participants were a group of highly selected patients. The generalizability of our findings to patients with significant comorbidities who might be considered for intensive hemodialysis in clinical practice remains uncertain.

In this analysis of the FHN Daily and Nocturnal trials, we found that RKF did not significantly influence LVM at baseline or its response to frequent hemodialysis. While frequent in-center hemodialysis was associated with significant reductions in LVM compared with conventional hemodialysis, this effect was not modified by baseline RKF status or urine volume. Neither baseline anuric status nor changes in urine volume over the study period were correlated with LVM changes. Therefore, the beneficial effect of frequent hemodialysis on LVM seems to be independent of RKF status, suggesting that patients may benefit from intensified hemodialysis regimens regardless of their baseline urine output. Future studies with larger sample sizes and more nuanced assessments of RKF are needed to further explore the complex relationship between RKF, hemodialysis intensity, and cardiovascular outcomes in patients with ESKD.

## Supplementary Material

## Data Availability

Original data generated for the study are or will be made available in a repository subject to controlled access. Data Type: Clinical Trial Data. Repository Name: NIDDK Repository. Linkable Citation, such as a DOI or Weblink: https://doi.org/10.58020/bx72-p494, https://doi.org/10.58020/prfs-az64. https://repository.niddk.nih.gov/study/168, https://repository.niddk.nih.gov/study/44. Reason for Restriction: The data that support the findings of this study are derived from the Frequent Hemodialysis Network (FHN) Daily and Nocturnal Trials. These data are available from the National Institute of Diabetes and Digestive and Kidney Diseases (NIDDK) Central Repository. Access to the data requires an application and approval from the NIDDK. The authors do not have the authority to share the data directly.

## Declarative Statements

This study includes clinical experimentation and received Institutional Review Board or Ethics Committee approval. The need to obtain informed patient consent was waived. This study includes clinical experimentation and complies with the Declaration of Helsinki.

## Supplemental Material

Supplemental Table 1. Mean change in log(LVM) by RKF status across subgroups.

Supplemental Figure 1. The response of LVM to hemodialysis in (A) the combined (3/wk and 6/wk hemodialysis) arms of the Daily and Nocturnal trials (*P* = 0.19 for between-group difference) and (B) in each arm of the Daily and Nocturnal trials.
