# Supplementary material for: Residual Kidney Function and Response of Left Ventricular Mass to Intensive Hemodialysis: The Frequent Hemodialysis Network Trials
Source: Kidney360. 2025 Sep 23;7(2):344–52. doi: 10.34067/KID.0000000987 (PMC12935374; doi:10.34067/KID.0000000987)
Supplement: Supplementary file 1 [file kidney360-7-344-s001.pdf]

ASN American Society of Nephrology | JASN Journal of the American Society of Nephrology | KIDNEY360 Accessing Our World From Every Angle | CJASN Clinical Journal of the American Society of Nephrology

ASN Journal Disclosure Form

As per ASN journal policy, I have disclosed any financial relationships or commitments I have held in the past 36 months as included below. I have listed my Current Employer below to indicate there is a relationship requiring disclosure. If no relationship exists, my Current Employer is not listed.

A. Kalogeropoulos reports the following:
Employer: Stony Brook University

I understand that the information above will be published within the journal article, if accepted, and that failure to comply and/or to accurately and completely report the potential financial conflicts of interest could lead to the following: 1) Prior to publication, article rejection, or 2) Post-publication, sanctions ranging from, but not limited to, issuing a correction, reporting the inaccurate information to the authors' institution, banning authors from submitting work to ASN journals for varying lengths of time, and/or retraction of the published work.

Name: Andreas P. Kalogeropoulos
Manuscript ID: K360-2025-000622R1
Manuscript Title: Residual Kidney Function and Response of Left Ventricular Mass to Intensive Hemodialysis The Frequent Hemodialysis Network Trials
Date of Completion: July 31, 2025
Disclosure Updated Date: July 31, 2025

ASN American Society of Nephrology JASN Journal of the American Society of Nephrology KIDNEY360 Accessing Our World From Every Angle CJASN Clinical Journal of the American Society of Nephrology

ASN Journal Disclosure Form

As per ASN journal policy, I have disclosed any financial relationships or commitments I have held in the past 36 months as included below. I have listed my Current Employer below to indicate there is a relationship requiring disclosure. If no relationship exists, my Current Employer is not listed.

S. Khan has nothing to disclose.

I understand that the information above will be published within the journal article, if accepted, and that failure to comply and/or to accurately and completely report the potential financial conflicts of interest could lead to the following: 1) Prior to publication, article rejection, or 2) Post-publication, sanctions ranging from, but not limited to, issuing a correction, reporting the inaccurate information to the authors' institution, banning authors from submitting work to ASN journals for varying lengths of time, and/or retraction of the published work.

Name: Sobia N. Khan
Manuscript ID: K360-2025-000622R1
Manuscript Title: Residual Kidney Function and response of Left ventricular mass to intensive hemodialysis The frequent Hemodialysis Network Trials
Date of Completion: July 31, 2025
Disclosure Updated Date: July 31, 2025

ASN American Society of Nephrology | JASN Journal of the American Society of Nephrology | KIDNEY360 Accessing Our World From Every Angle | CJASN Clinical Journal of the American Society of Nephrology

ASN Journal Disclosure Form

As per ASN journal policy, I have disclosed any financial relationships or commitments I have held in the past 36 months as included below. I have listed my Current Employer below to indicate there is a relationship requiring disclosure. If no relationship exists, my Current Employer is not listed.

S. Mallipattu reports the following:
Employer: Stony Brook Medicine; Consultancy: Wildwood Therapeutics, Inc.; L.E.K. Consulting; Dedham Group; Graticule;; Research Funding: Dialysis Clinic Inc.; Patents or Royalties: Krüppel-like factor 15 (KLF15) Small Molecule Agonists in Kidney Disease. US 63/018.247. 2023.; and Advisory or Leadership Role: Clinically Integrated Network, Board Member (Accountable Care Organization, LLC Stony Brook Medicine);.

I understand that the information above will be published within the journal article, if accepted, and that failure to comply and/or to accurately and completely report the potential financial conflicts of interest could lead to the following: 1) Prior to publication, article rejection, or 2) Post-publication, sanctions ranging from, but not limited to, issuing a correction, reporting the inaccurate information to the authors' institution, banning authors from submitting work to ASN journals for varying lengths of time, and/or retraction of the published work.

Name: Sandeep K. Mallipattu
Manuscript ID: K360-2025-000622R1
Manuscript Title: Residual Kidney Function and Response of Left Ventricular Mass to Intensive Hemodialysis The Frequent Hemodialysis Network Trials
Date of Completion: August 1, 2025
Disclosure Updated Date: March 18, 2025

ASN American Society of Nephrology | JASN Journal of the American Society of Nephrology | KIDNEY360 Accessing Our World From Every Angle | CJASN Clinical Journal of the American Society of Nephrology

ASN Journal Disclosure Form

As per ASN journal policy, I have disclosed any financial relationships or commitments I have held in the past 36 months as included below. I have listed my Current Employer below to indicate there is a relationship requiring disclosure. If no relationship exists, my Current Employer is not listed.

T. Shafi reports the following:
Employer: Baylor Scott & White Health; and Consultancy: Allucent (DSMB Member), Nephrolyx, Petauri Kinect.

I understand that the information above will be published within the journal article, if accepted, and that failure to comply and/or to accurately and completely report the potential financial conflicts of interest could lead to the following: 1) Prior to publication, article rejection, or 2) Post-publication, sanctions ranging from, but not limited to, issuing a correction, reporting the inaccurate information to the authors' institution, banning authors from submitting work to ASN journals for varying lengths of time, and/or retraction of the published work.

Name: Tariq Shafi
Manuscript ID: K360-2025-000622R1
Manuscript Title: Residual Kidney Function and Response of Left Ventricular Mass to Intensive Hemodialysis The Frequent Hemodialysis Network Trials
Date of Completion: July 31, 2025
Disclosure Updated Date: July 31, 2025

ASN
American Society of Nephrology
JASN
Journal of the American Society of Nephrology
KIDNEY360
Accessing Our World From Every Angle
CJASN
Clinical Journal of the American Society of Nephrology

ASN Journal Disclosure Form

As per ASN journal policy, I have disclosed any financial relationships or commitments I have held in the past 36 months as included below. I have listed my Current Employer below to indicate there is a relationship requiring disclosure. If no relationship exists, my Current Employer is not listed.

A. Tajerian has nothing to disclose.

I understand that the information above will be published within the journal article, if accepted, and that failure to comply and/or to accurately and completely report the potential financial conflicts of interest could lead to the following: 1) Prior to publication, article rejection, or 2) Post-publication, sanctions ranging from, but not limited to, issuing a correction, reporting the inaccurate information to the authors' institution, banning authors from submitting work to ASN journals for varying lengths of time, and/or retraction of the published work.

Name: Amin Tajerian
Manuscript ID: K360-2025-000622R2
Manuscript Title: Residual Kidney Function and Response of Left Ventricular Mass to Intensive Hemodialysis The Frequent Hemodialysis Network Trials
Date of Completion: August 26, 2025
Disclosure Updated Date: August 26, 2025

ASN American Society of Nephrology | JASN Journal of the American Society of Nephrology | KIDNEY360 Accessing Our World From Every Angle | CJASN Clinical Journal of the American Society of Nephrology

ASN Journal Disclosure Form

As per ASN journal policy, I have disclosed any financial relationships or commitments I have held in the past 36 months as included below. I have listed my Current Employer below to indicate there is a relationship requiring disclosure. If no relationship exists, my Current Employer is not listed.

J. Zhang has nothing to disclose.

I understand that the information above will be published within the journal article, if accepted, and that failure to comply and/or to accurately and completely report the potential financial conflicts of interest could lead to the following: 1) Prior to publication, article rejection, or 2) Post-publication, sanctions ranging from, but not limited to, issuing a correction, reporting the inaccurate information to the authors' institution, banning authors from submitting work to ASN journals for varying lengths of time, and/or retraction of the published work.

Name: Jason Zhang
Manuscript ID: K360-2025-000622R2
Manuscript Title: Residual Kidney Function and Response of Left Ventricular Mass to Intensive Hemodialysis The Frequent Hemodialysis Network Trials
Date of Completion: August 21, 2025
Disclosure Updated Date: August 1, 2025
