# Supplementary material for: Residual Kidney Function and Response of Left Ventricular Mass to Intensive Hemodialysis: The Frequent Hemodialysis Network Trials
Source: Kidney360. 2025 Sep 23;7(2):344–52. doi: 10.34067/KID.0000000987 (PMC12935374; doi:10.34067/KID.0000000987)
Supplement: Supplementary file 2 [file kidney360-7-344-s002.pdf]

Table of Contents

- **Supplemental Table 1**
- **Supplemental Figure 1**

Supplemental Table 1: Mean Change in log(LVM) by RKF Status Across Subgroups

**Subgroup**	**RKF+ (≥100 ml/day)**	**RKF– (<100 ml/day)**	**p-value**
**Race**			
Black	–0.06 ± 0.19	–0.07 ± 0.22	0.667
Other or Unknown	–0.05 ± 0.24	–0.03 ± 0.22	0.626
White	–0.05 ± 0.21	–0.08 ± 0.23	0.517
**Gender**			
Female	–0.01 ± 0.24	–0.07 ± 0.23	0.195
Male	–0.08 ± 0.19	–0.06 ± 0.22	0.615
**Trial Group**			
Daily (in-center)	–0.07 ± 0.21	–0.07 ± 0.23	0.941
Nocturnal (home)	–0.04 ± 0.21	–0.02 ± 0.19	0.823
**Ethnicity**			
Hispanic	–0.12 ± 0.22	–0.05 ± 0.24	0.316
Non-Hispanic	–0.04 ± 0.21	–0.07 ± 0.22	0.383
**Dialysis Treatment Type**			
Intensive Hemodialysis (6x/week)	–0.08 ± 0.23	–0.11 ± 0.23	0.437
Conventional Hemodialysis (3x/week)	–0.03 ± 0.18	0.00 ± 0.19	0.434

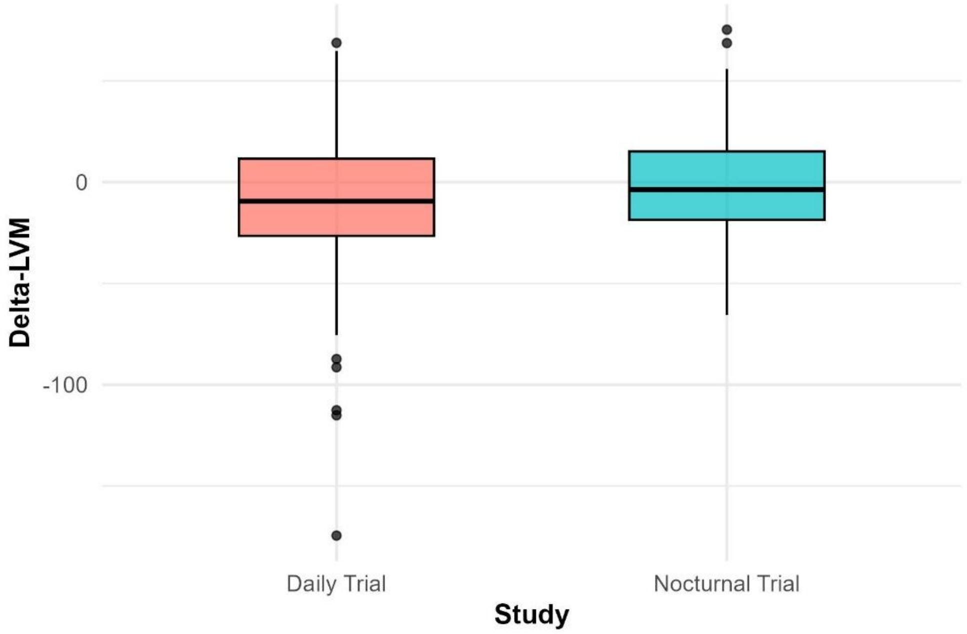

a.

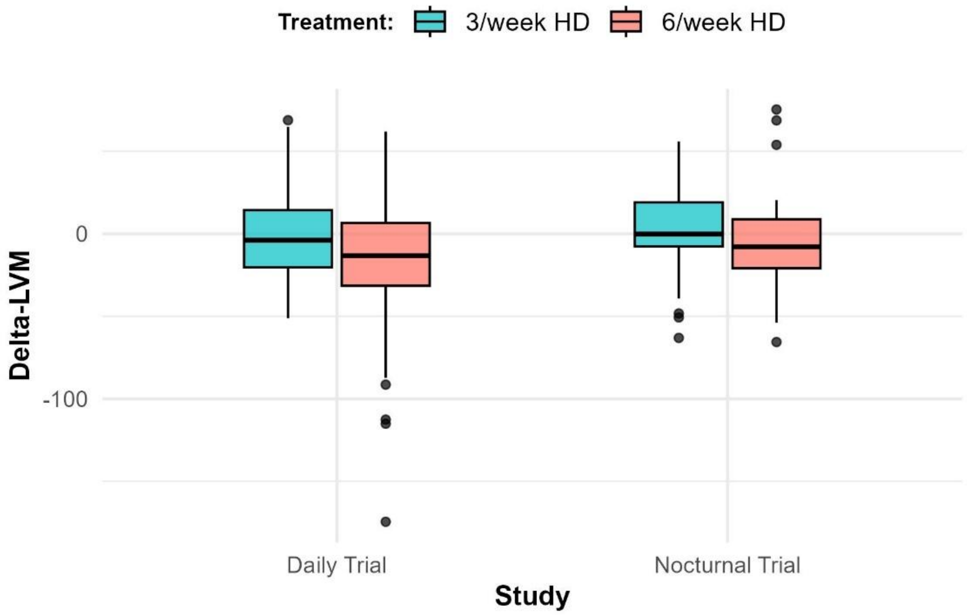

b.

Supplemental Figure 1. The response of LVM to HD in (A) the combined (3/week and 6/week HD) arms of the Daily and Nocturnal trials (P=0.19 for between-group difference) and (B) in each arm of the Daily and Nocturnal trials. Between group differences: P=0.14 in the Daily trial; P=0.18 in the Nocturnal trial.
